# *Kcne4* deletion sex-specifically predisposes to cardiac arrhythmia via testosterone-dependent impairment of RISK/SAFE pathway induction in aged mice

**DOI:** 10.1038/s41598-018-26599-8

**Published:** 2018-05-29

**Authors:** Zhaoyang Hu, Wei Wei, Leng Zhou, Mou Chen, Geoffrey W. Abbott

**Affiliations:** 1 Laboratory of Anesthesiology & Critical Care Medicine, Translational Neuroscience Center, West China Hospital, Sichuan University, Chengdu, Sichuan China; 2 Department of Anesthesiology, West China Hospital, Sichuan University, Chengdu, Sichuan China; 3 Bioelectricity Laboratory, Dept. of Physiology and Biophysics, School of Medicine, University of California, Irvine, CA USA

## Abstract

Sudden cardiac death (SCD) is associated with both electrical and ischemic substrates, and is a major cause of ischemic heart disease mortality worldwide. Male sex predisposes to SCD but the underlying mechanisms are incompletely understood. KCNE4, a cardiac arrhythmia-associated potassium channel β-subunit, is upregulated by 5α-dihydrotestosterone (DHT). Thus, ventricular *Kcne4* expression is low in young adult female mice, but high in males and postmenopausal (12+ months) females. Despite causing a sex-independent electrical substrate at 13 months of age (22% QT prolongation in both males and females; P < 0.01), *Kcne4* deletion preferentially predisposed aged male mice to ischemia/reperfusion (IR)-provoked ventricular tachyarrhythmias. Interestingly, *Kcne4* deletion caused baseline induction of cardioprotective RISK and SAFE pathways in 13-m-old female, but not male, mice. IR-invoked RISK/SAFE induction was also deficient in male but not female *Kcne4*^−/−^ mice. Pharmacological inhibition of RISK/SAFE pathways in *Kcne4*^−/−^ females eliminated sex-specific differences in IR-invoked tachyarrhythmia predisposition. Furthermore, castration of *Kcne4*^−/−^ males eliminated sex-specific differences in both baseline and post-IR RISK/SAFE pathway induction, and tachyarrhythmia predisposition. Our results demonstrate for the first time that male sex can predispose in aged mice to dangerous ventricular tachyarrhythmias despite sex-independent electrical and ischemic substrates, because of testosterone-dependent impairment of RISK/SAFE pathway induction.

## Introduction

Sudden cardiac death (SCD) accounts for 1000 deaths per day in the United States alone. SCD is thought to require electrical and ischemic substrates, and a trigger (commonly premature ventricular complexes or PVCs)^1^. Monogenic inherited forms of SCD are almost exclusively linked to genes encoding ion channels or the proteins that regulate them^2^. Inherited ventricular arrhythmias that predispose to SCD include Long QT syndrome (LQTS), observed as prolongation of the QT interval on the body surface electrocardiogram (ECG). The most common inherited forms of LQTS (>80% of sequenced cases) are caused by loss-of-function mutations in either of the main ventricular myocyte repolarizing voltage-gated potassium (Kv) channels, KCNQ1 (Kv7.1) and hERG (KCNH2; Kv11.1), while a further 10% of cases are caused by gain-of-function mutations in *SCN5A*, encoding the Nav1.5 voltage-gated sodium channel^3^. The rest primarily involve regulators of ion channels, including the *KCNE* genes, of which there are five in the human genome^2,4^. *KCNE* genes encode single-transmembrane-domain subunits that form complexes with Kv α subunits, and also α subunits of HCN and L-type Ca^2+^ channels, to alter their functional properties. These properties include anterograde and retrograde trafficking, subunit composition, gating kinetics and voltage dependence, conductance, ion selectivity, regulation by other molecules and proteins, and pharmacology^5,6^.

KCNE4 is the largest of the KCNE subunits (human short isoform, 170 amino acids; long isoform, 221)^6^ and the most highly expressed in human heart (both atria and ventricles) according to two independent real-time qPCR analyses^7,8^. KCNE4 regulates a variety of Kv channels, including cardiac-expressed KCNQ1^9,10^, Kv1.5 and Kv2.1 (which generate *I*_K,slow_ in mice)^11,12^, and Kv4.2 and Kv4.3 (which generate the transient outward current, *I*_to_)^6,13^; depending on the species, some or all of these channels are regulated by KCNE4 in cardiac myocytes. In addition, KCNE4 forms complexes and regulates the voltage dependence and protein stability of BK (Ca^2+^-activated) K^+^ channels in the kidney^14^, KCNQ4 in the vasculature^6,15,16^, and Kv1.1 and Kv1.3^17^.

Human SCD exhibits marked sex-specific features, with men showing increased predisposition to SCD compared to women^18^. However, the molecular basis for this sex-dependence has not been explained. We previously found that cardiac KCNE4 expression is positively regulated by 5α-dihydrotestosterone (DHT) and that *Kcne4* deletion in mice age- and sex-specifically impairs ventricular repolarization, with both young and old males being affected, while female mice were only affected following menopause (>12 months), when their DHT levels rose. This tallied well with a relative paucity of cardiac *Kcne4* expression in young adult female mice, compared to young and old males and postmenopausal females^11^. Because of the aforementioned male predisposition to SCD, and the requirement in SCD of both an electrical and an ischemic substrate, here we asked whether deletion of *Kcne4* in mice sex-specifically affects arrhythmia predisposition, before versus after coronary artery ligation to mimic the cardiac ischemia that, together with an electrical substrate, is thought to predispose to SCD.

## Materials and Methods

### Animals

*Kcne4*^+/+^ and *Kcne4*^−/−^ C57BL/6 mice were generated as previously described^11^ and housed and used according to the recommendations in the Guide for the Care and Use of Laboratory Animals of the National Institutes of Health (8th edition, 2011). The study was approved by the Institutional Animal Care and Use Committee of Sichuan University (Sichuan, China) (Permit Number: 2015033 A). Adult male and female mice of 13 months of age, generated from *Kcne4*^+/+^ × *Kcne4*^−/−^ crosses, were used.

### Surgical procedures

Details of the surgical procedures have been described previously^19,20^. Briefly, sodium pentobarbital (50 mg/kg, ip) was used for anesthesia. Baseline ECG values were recorded 10 minutes after stabilization. Mice were intubated with an endotracheal tube (PE90) attached to a mouse ventilator (Harvard Apparatus, Holliston, MA). Mice were ventilated with a tidal volume of 250 μL at a rate of 150 strokes/min.

To assess the role of KCNE4 in cardiac ischemia/reperfusion (IR)-induced ventricular arrhythmias, the chest was opened and the heart was exposed. The main left coronary artery was identified and a 9-0 silk ligature (Ethicon, Somerville, NJ, USA) was placed close to its origin for production of coronary artery occlusion and reperfusion. After ten minutes of ligation, the suture was released followed by 20 minutes of reperfusion. This stringent but relatively brief IR injury protocol is designed to induce acute ischemia-associated arrhythmias, not an extensive infarct. Successful ligation was verified by visualizing a regional dyskinesia and epicardial cyanosis in the ischemic zone. Reperfusion was confirmed by the presence of an epicardial hyperemic response. Anesthesia adequacy was controlled by monitoring the lack of response to toe-pinching and the loss of the corneal reflex. It was then monitored by evaluating heart rate throughout the experiment. Where appropriate, 30 minutes prior to left main coronary artery ligation, pharmacological inhibitors including AKT inhibitor wortmannin (15 µg/kg) (Sigma, St. Louis, MO, USA), ERK1/2 inhibitor U0126 (0.5 mg/kg) (Sigma),or STAT-3 inhibitor Ag490 (5 mg/kg) (Sigma) were intravenously bolus-injected into the female femoral vein. Mice body temperature was maintained with a heating lamp. Male *Kcne4*^+/+^ and *Kcne4*^−/−^ mice (8–10 months old) were castrated to establish a testosterone deficiency model. Three months after castration, left ventricles were harvested for western blotting. IR surgery was used to induce ventricular arrhythmias. At the end of the reperfusion, mice were euthanized with an overdose of sodium pentobarbital (200 mg/kg, i.p.) and death was monitored by cardiac activity and respiration. The coronary artery was then re-occluded followed by injection of 1% Evans blue (Sigma, St. Louis, MO, USA) into the left ventricular cavity to depict the ischemic area at risk (AAR) within the left ventricle. AAR was isolated and stored at −80 °C for protein phosphorylation analysis.

### Electrocardiography analysis

ECG studies were performed using PowerLab/8sp and LabChart 7.2.1 software (AD Instruments, Colorado Springs, CO, USA). Mice were anesthetized with intraperitoneal injection of sodium pentobarbital (50 mg/kg) and placed in a supine position. After anesthesia, a standard limb lead II configuration electrocardiographic system was subcutaneously attached to the mice underneath the skin by needle electrodes. Baseline ECG parameters included: RR intervals (RR), heart rate (HR), PR intervals (PR), QRS intervals (QRS), QT intervals (QT), corrected QT interval (QTc), and ST-segment (the period between the end of the QRS complex and the beginning of the T wave). QTc was calculated based on Mitchell’s formula specifically for mice: QTc = QT/(RR/100)^1/2 ^^21^.

### Tissue collection

After sacrificing the mice, the hearts were dissected and the atria removed. The ventricles were then rinsed in saline, dried, embedded in 10% phosphate-buffered formalin overnight before cutting into 5-μm sections parallel to the atrioventricular groove. Serial sections of transverse myocardial slices were deparaffinized in xylene and isopropanol and then were mounted on glass slides for hematoxylin and eosin stain or terminal deoxynucleotidyl transferase-mediated dUTP nick-end labeling (TUNEL) staining.

### Histological evaluation of edema

In the baseline condition hearts, edema was the only gross pathology observed. A modified numerical scoring system was used in this analysis for histological evaluation based on a scoring system we described previously^22^. Briefly, the degree of myocardial edema was graded according to severity (0: no edema, 1: mild, 2: moderate, and 3: marked) and distribution (0: no edema, 1: focal edema, 2: multifocal edema, and 3: diffuse edema). A mean score for each variable was determined for each heart. Hearts were evaluated in a double-blind manner.

### TUNEL staining

TUNEL assay was applied for determination of cardiac cell apoptosis according to the manufacturer’s protocol (Roche Diagnostics, Indianapolis, IN, USA). Ten fields from each heart were chosen randomly and analyzed in a blinded manner. TUNEL-positive apoptotic nuclei were stained red and TUNEL-negative nuclei were stained blue. The apoptotic index was calculated as a ratio of the number of TUNEL-positive nuclei to the total nuclei population. Images were obtained with an inverted microscope (Olympus A/S, Ballerup, Denmark).

### Arrhythmia analysis

Arrhythmia events were continuously recorded throughout the entire cardiac ischemia and reperfusion injury period. Arrhythmia parameters were analyzed offline using LabChart7.2.1 software (AD Instruments, Colorado Springs, CO, USA): number of mice exhibiting (1) cardiac arrhythmia; (2) AV block (AVB); (3) sudden cardiac death (SCD) during the 20 min reperfusion period; (4) ventricular tachycardia (VT) (5) polymorphic VT (PVT); (6) sustained ventricular tachycardia over 20 seconds (SVT); (7) VT duration (8) the latency of VT.

### Testosterone measurements

Serum quantification of total testosterone was measured for each mouse tested in duplicate via ELISA according to the manufacturer’s instructions (Nanjing Jiancheng Bioengineering Institute, Nanjing, China).

### Western blotting

Frozen myocardial tissue samples were homogenized with a precooled pestle grinder system on ice (Fisher Scientific, Hampton, NH, USA) in ice-cold RIPA buffer containing 50 mM Tris-HCl (pH7.4), 150 mM NaCl, 1% NP-40, 1 mM EDTA, 0.25% sodium deoxycholate, mixed with phosphatase inhibitor cocktail (Sigma-Aldrich, St. Louis, MO, USA), and a protease inhibitor cocktail (Sigma-Aldrich, St. Louis, MO, USA). The homogenate was then centrifuged at 10,000 g for 10 min at 4 °C and the resulting supernatant was collected. Protein concentration was determined by BCA method according to the manufacturer’s instructions. (Pierce, Rockford, IL, USA). Aliquots of the supernatant containing equal amounts of protein (15 μg) dissolved in sample buffer (Bio-Rad, Hercules, CA, USA) were heated at 95 °C for 10 min before resolving on a 12% SDS-PAGE gel. Proteins were then transferred onto nitrocellulose membranes (VWR, Batavia, IL, USA) and blocked with non-fat milk for 1 hour at room temperature.

Primary rabbit antibodies included phosphorylated extracellular signal-regulated kinase 1/2 (ERK1/2)(Thr202/Tyr204) (p-ERK), total ERK1/2, phosphorylated Akt(ser473) (p-AKT), total Akt, phosphorylated glycogen synthase kinase-3β(Ser9) (p-GSK-3β), total -GSK-3β, phosphorylated STAT-3(Tyr705) (p-STAT-3) and total STAT-3 (all, 1:1000, Cell Signaling, Danvers, MA, USA). After overnight incubation of primary antibodies, horseradish peroxidase (HRP)-conjugated goat anti-rabbit IgG secondary antibody (1:5000, Bio-Rad, Hercules, CA, USA) was used. Immunoreactive bands were then detected by chemiluminescence western blotting ECL (Millipore, Billerica, MA, USA). Signals were obtained using an AmershamImager 600 system (GE Healthcare, Little Chalfont, UK) and band densities were determined by ImageJ Data Acquisition Software (National Institutes of Health, Bethesda, MD, USA). Phosphorylation signal densities were normalized to total protein-signal densities. Raw western blot images showing entire blot are included in Supplementary Information.

### Statistical analysis

All values except percentages are expressed as mean ± SEM. The assumption of normal distribution was confirmed by the Kolmogorov-Smirnov test. The unpaired two-tailed student’s t-tests were used for two values comparisons. Fisher’s exact test was used to compare numbers of mice assigned into one of two groups. One-way ANOVA was applied for multiple comparisons over three groups. Levene’s test was used to test homogeneity of variance. If variances were equal, the Newman-Keuls test was examined post hoc for multiple comparisons; otherwise, Dunnett’s T3 test was applied. Two-way repeated-measures ANOVA was used to assess the changes of ST height during the coronary artery occlusion period. Sphericity assumption was determined by Mauchly’s test. If not applicable, a Greenhouse–Geisser correction was applied for degrees-of-freedom adjustment. When differences were detected between groups, the Bonferroni correction procedure was used for multiple comparisons; otherwise, global conclusions were drawn. All P-values were two-sided. Statistical significance was defined as P < 0.05.

### Data Availability

All data generated or analyzed during the current study are included in this published article (and its Supplementary Information file). Materials will be made available to others upon reasonable request.

## Results

### Kcne4 deletion sex-independently causes QT prolongation in aging mice

**Figure 1:**
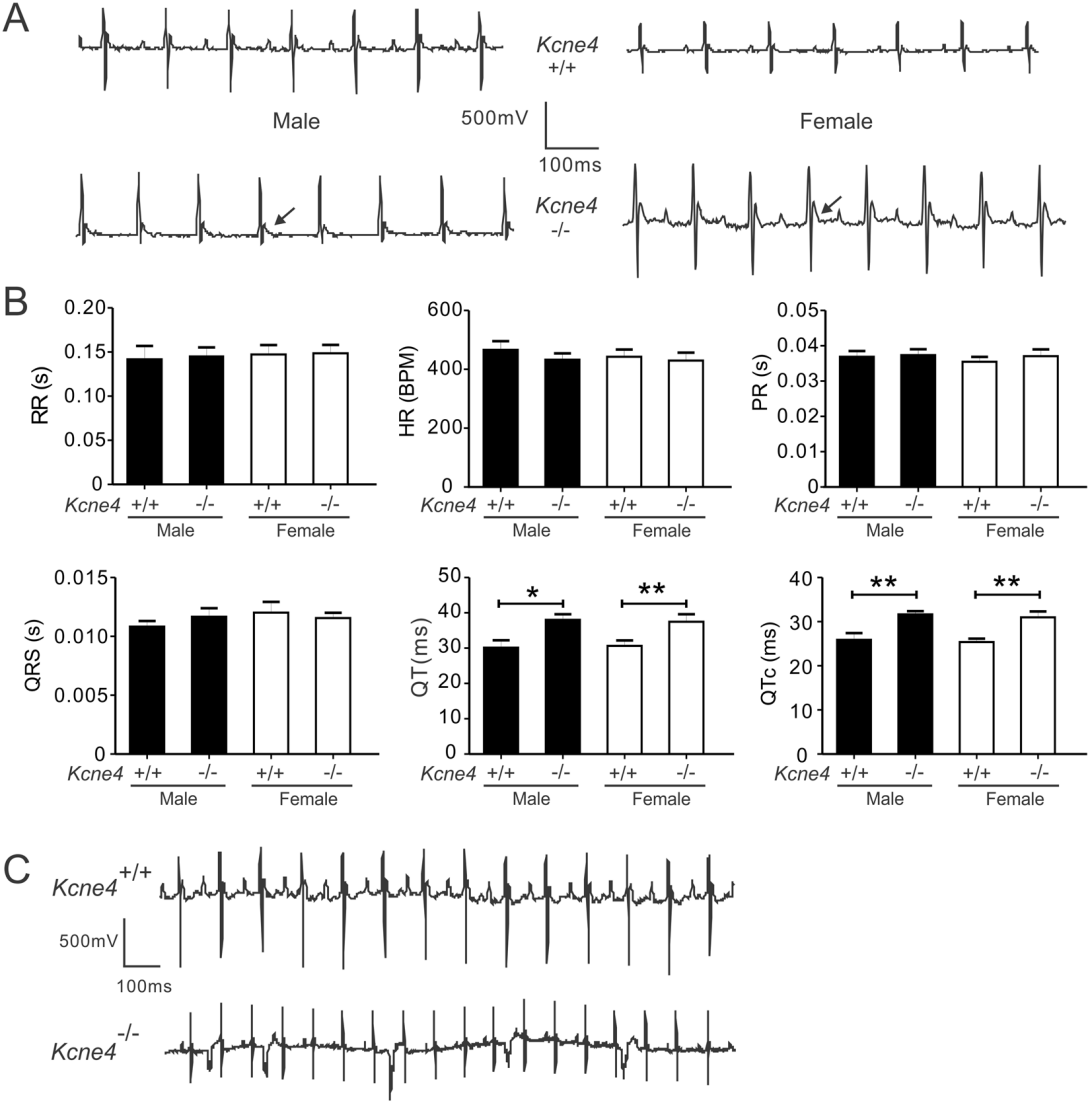
Kcne4 deletion sex-independently delays ventricular repolarization in aging mice. (**A**) Exemplar surface ECGs from male and female *Kcne4*^+/+^ and *Kcne4*^−/−^ mice showing QT prolongation (arrow) in the latter. (**B**) Quantification of parameters from electrocardiograms as in A showing QT and QTc prolongation in *Kcne4*^−/−^ mice of either sex (*n* = 12–18). *P < 0.05, **P < 0.01 between genotypes. All other group comparisons *P* > 0.05. RR, RR interval; HR, heart rate; PR, PR interval; QRS, QRS interval. (**C**) Exemplar surface ECG traces belonging to the only male *Kcne4*^−/−^ mouse exhibiting frequent ventricular premature beats (VPBs).

We previously found that aging male and female *Kcne4*^−/−^ mice have similar QT prolongation, but we checked again to determine if this held true in our current mouse colony at the ages studied herein. Consistent with our previous findings^11^, *Kcne4* deletion caused QTc prolongation equally in aging mice of both sexes; compared with wildtype littermates, QT and QTc were increased by 26.0% (P < 0.05) and 22.2% (P < 0.01) in male *Kcne4*^−/−^ mice, and by 22.4% (P < 0.01) and 22.0% (P < 0.01) in female *Kcne4*^−/−^ mice, respectively. Other electrocardiographic parameters such as heart rate, PR, RR and QRS intervals were not changed in either sex (Fig. 1A,B). Ventricular premature beats (VPB), a cause of ventricular tachycardia or ventricular fibrillation, can arise from QT prolongation and can ultimately lead to SCD. We observed, only in one male *Kcne4*^−/−^ mouse, VPB occurring in ~25% of beats (Fig. 1C); VPBs were not observed in any of the female mice or wild-type male mice. Thus, aside from VPB in one male *Kcne4*^−/−^ mouse, we observed similar QTc prolongation and other electrocardiographic parameters at baseline in aging male and female *Kcne4*^−/−^ mice.

### Kcne4 deletion sex-dependently predisposes to reperfusion-induced arrhythmias

**Figure 2:**
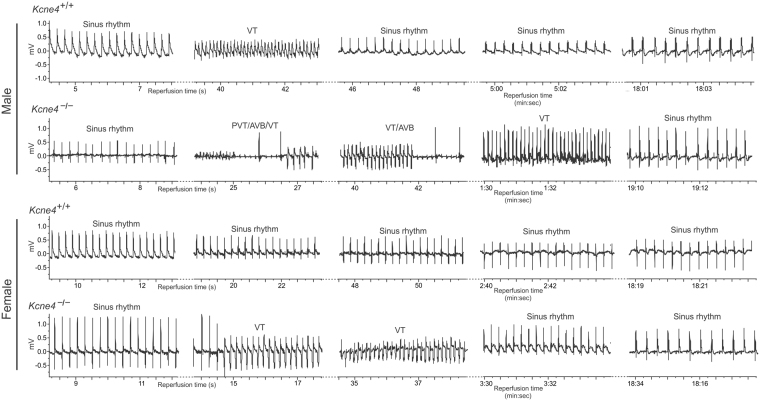
Male sex exacerbates effects of Kcne4 deletion on predisposition to post-IR arrhythmias. Exemplar surface ECGs of *Kcne4*^+/+^ and *Kcne4*^−/−^ mice of either sex during the cardiac reperfusion period (*n* = 10–15). PVT indicates polymorphic VT; VT, ventricular tachycardia; AVB, AV block.

**Figure 3:**
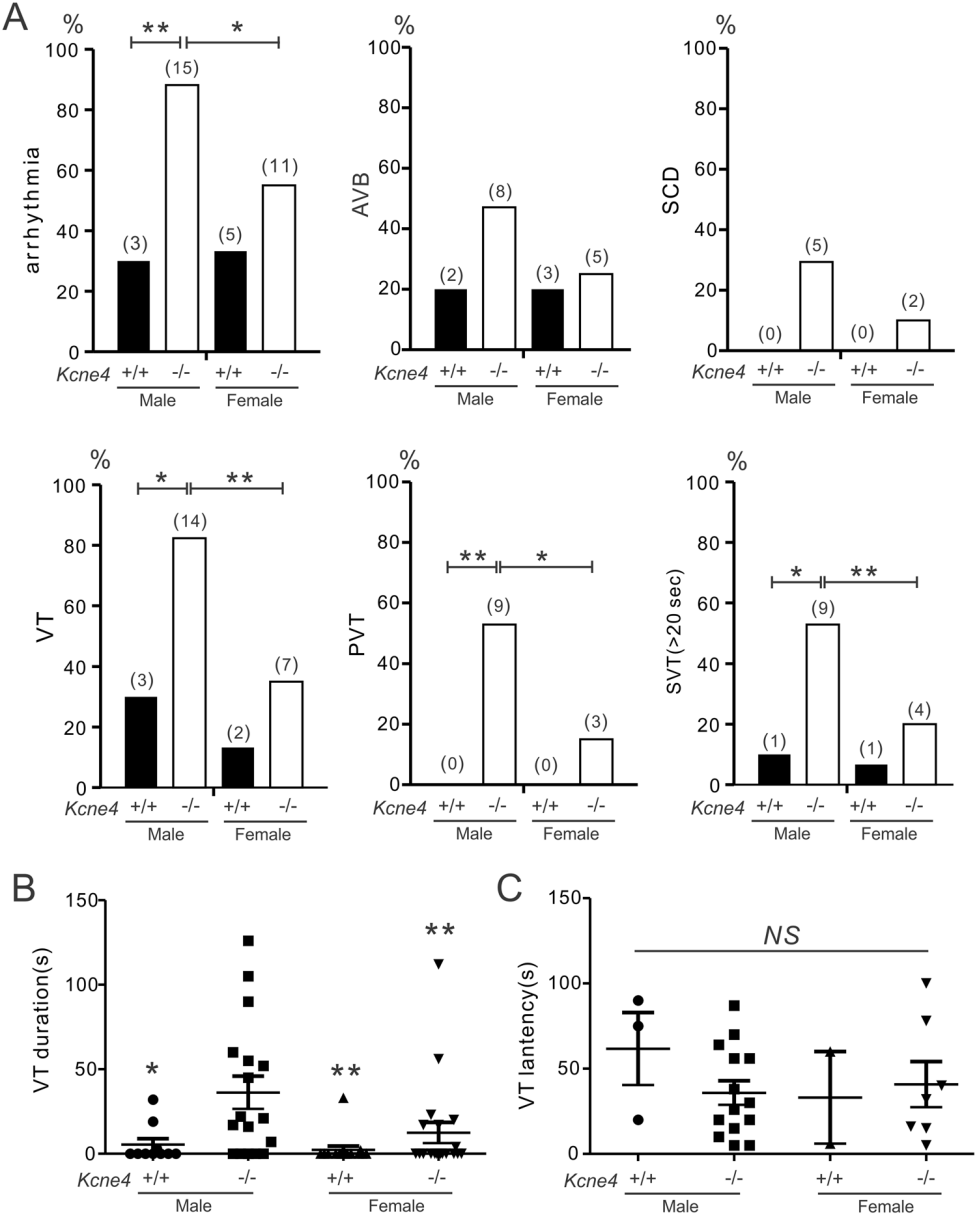
Male Kcne4^−/−^ mice are especially predisposed to longer and more severe post-IR ventricular tachyarrhythmias. (**A**) Quantification of cardiac arrhythmia incidence during post-ischemia reperfusion in male *Kcne4*^+/+^ (*n* = 10) and *Kcne4*^−/−^ (*n* = 17) and female *Kcne4*^+/+^ (*n* = 15) and *Kcne4*^−/−^ (*n* = 20) mice. Numbers of mice exhibiting each arrhythmia category are indicated in parentheses above columns. AVB, AV block; SCD, sudden cardiac death; VT, ventricular tachycardia; PVT, polymorphic VT; SVT, sustained ventricular tachycardia over 20 seconds. *P < 0.05; **P < 0.01 between genotypes or sexes. (**B**) Mean VT durations for male and female *Kcne4*^+/+^ and *Kcne4*^−/−^ mice; *n* as in panel A. Mice without VT were indicated as 0 s duration. *P < 0.05, **P < *0.01*, vs. male *Kcne4*^−/−^ mice (by one-way ANOVA). (**C**) Latency to first run of VT after the onset of reperfusion in male and female *Kcne4*^+/+^ and *Kcne4*^−/−^ mice; *n* as in panel A. NS, *P* > *0.05* among groups (by one-way ANOVA).

We next quantified the effects of *Kcne4* deletion on arrhythmia predisposition in the context of an ischemic substrate (IR injury by coronary artery ligation), mimicking the conditions thought to be required for SCD. Representative ECG tracings from *Kcne4*^+/+^ and *Kcne4*^−/−^ mice of either sex during post-ischemic reperfusion are presented in Fig. 2, and quantification shown in Fig. 3. Classes of post-IR arrhythmic events included atrioventricular block (AVB), sudden cardiac death (SCD), ventricular tachycardia (VT), polymorphic ventricular tachycardia (PVT), and sustained ventricular tachycardia (>20 seconds) (SVT).

During the cardiac reperfusion period, 7/10 (70%) male *Kcne4*^+/+^ mice remained in sinus rhythm, compared to only 2 out of 17 (12%) male *Kcne4*^−/−^ mice (P = 0.004) (Fig. 3A). In female mice, *Kcne4* deletion was not as effective at increasing reperfusion arrhythmia incidence (P = 0.31). There was a similar pattern for AVB induction and for SCD specifically, although none of the changes (male or female) reached statistical significance (P > 0.05). *Kcne4* deletion was much more effective at inducing reperfusion VT, PVT, and SVT in males than in female mice; for example, only 15% of female *Kcne4*^−/−^ mice (3/20) developed polymorphic VT compared to 53% of male *Kcne4*^−/−^ mice (9/17). SVT was 5-fold more common in male *Kcne4*^−/−^ mice (9/17) than male *Kcne4*^+/+^ mice (1/10) (P = 0.04), whereas female *Kcne4*^−/−^ mice (4/20) had a similarly low incidence of SVT as their wildtype littermates (1/15) (P = 0.4). Additionally, mean VT duration was 3-fold longer in male *Kcne4*^−/−^ mice (36.2 ± 9.7 s) compared to female *Kcne4*^−/−^ mice (12.5 ± 6.1 s, P = 0.003 by one-way ANOVA, Fig. 3B), while the latency to first recorded VT episode was similar in either group (P = 0.5, Fig. 3C).

### Kcne4 deletion stimulates baseline ERK1/2, AKT, and STAT-3 phosphorylation in aging female but not male mice

**Figure 4:**
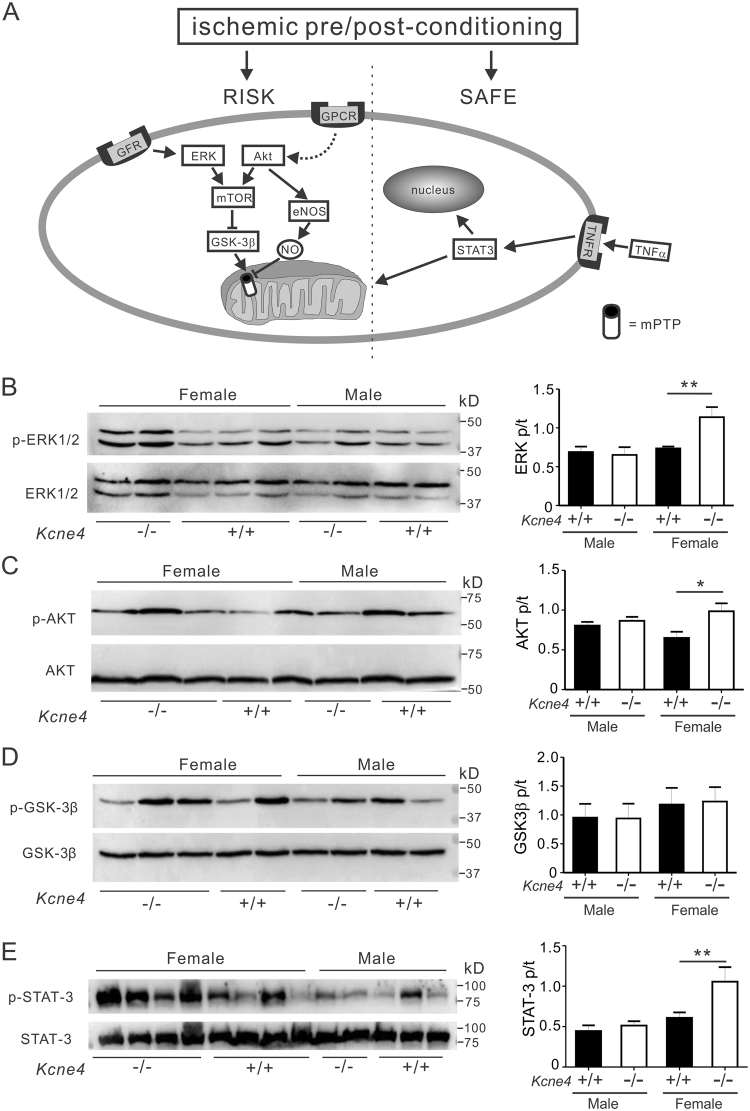
Kcne4 deletion activates RISK and SAFE pathways at baseline in female mice. (**A**) Schematic of the RISK and SAFE cardioprotective pathways. mPTP, mitochondrial permeability transition pore. (**B**–**E**) *Left*, representative western blots of phospho-(p) ERK1/2 and total (t) ERK1/2 (**B**), phospho-(p) AKT and total (t) AKT (**C**), phospho-(p) GSK3β and total (t) GSK3β (**D**), phospho-(p) STAT-3 and total (t) STAT-3 (**E**) from baseline male and female *Kcne4*^+/+^ and *Kcne4*^−/−^ ventricles; one mouse per lane. *Right*, mean ratio of pERK/tERK (**B**, *n* = 5–7), pAKT/tAKT (**C**, *n* = 5–7), pGSK3β/tGSK3β (**D**, *n* = 7–10), pSTAT-3/tSTAT-3 (**E**, *n* = 5–9) band densities from blots as in left. *P < 0.05, **P < *0.01*, between genotypes.

Given that *Kcne4* deletion sex-independently prolonged the QT interval by 22% in both male and female mice, but predisposed to worse ventricular tachyarrythmias in male mice versus female mice, we sought a molecular basis for this discrepancy. The reperfusion injury salvage kinase (RISK) signaling pathway, which includes protein kinase B (AKT) and extracellular signal-regulated kinases (ERK1/2), is a fundamental signal transduction cascade in the cardioprotective mechanism of local or remote ischemic preconditioning^23^. Activation of the RISK pathway confers powerful cardioprotection against reperfusion injury. Ischemic conditioning stimuli leads to ERK1/2 or AKT phosphorylation and thus activation, and reduced infarct size^24^, as we previously observed for *Kcne2* null mice^22^. This protective phenomenon is mediated through inhibition of mitochondrial permeability transition pore (mPTP) opening and myocyte apoptosis^25^. Additionally, phosphorylation of GSK-3β(Ser9), a downstream target of AKT and ERK1/2, results in the inhibition of GSK-3β and enhancement of myocardial survival against IR^26^. Importantly, RISK pathway induction can also reduce the severity of IR-induced ventricular arrhythmias^27,28^. Independent of the RISK pathway, an alternative protective pathway was recently recognized and termed the survivor activating factor enhancement (SAFE) pathway. It includes signal transducer and activator of transcription 3 (STAT-3) and offers protection in ischemic conditioning^29^ (Fig. 4A). Inhibiting the SAFE pathway can also abolish the infarct-sparing effects of ischemic pre- or post-conditioning^30^.

We previously discovered baseline activation of the cardioprotective RISK pathway in *Kcne2* null mice, likely arising from the multiple systemic defects cause by *Kcne2* deletion^22^. Here, because KCNE4 is known to be expressed and to regulate physiologically important potassium channels in multiple tissues, including the heart, kidneys and vasculature, we assessed the effects of *Kcne4* deletion on constitutive activation of both RISK and SAFE pathways in ventricular tissue, at 13 months of age. In males, *Kcne4* deletion did not alter baseline phosphorylation of any of the four proteins tested. In contrast, we observed 55% increased ERK1/2 phosphorylation in female *Kcne4*^−/−^ ventricles (P < 0.01, Fig. 4B), while AKT phosphorylation was increased by 51% in female *Kcne4*^−/−^ ventricles (P < 0.05, Fig. 4C). The ratio of phosphorylated (p) to total (t) GSK-3β at baseline remained unchanged in either sex (P > 0.05, Fig. 4D), while phospho-STAT-3 levels were enhanced only in female *Kcne4*^−/−^ mice compared to female *Kcne4*^+/+^ mice (by 74%, P < 0.01, Fig. 4E). Thus, *Kcne4* deletion induces baseline RISK and SAFE pathway activation, only in female mice.

### Kcne4 deletion impairs post-ischemic RISK and SAFE pathway induction in male but not female mice

The baseline induction of cardioprotective pathways in female *Kcne4*^−/−^ mice was one plausible explanation for their tolerance to IR injury, but were there additional mechanisms underlying the increased IR injury-induced arrhythmia predisposition in *Kcne4*^−/−^ males versus females? We next performed histological analyses to uncover possible mechanisms, but found no other factors that might explain the males’ predisposition to *Kcne4*-linked reperfusion arrhythmia predisposition. Specifically, we did not detect increased myocardial edema, apoptosis or fibrosis in aging male *Kcne4*^−/−^ hearts compared to those of age-matched wild-type littermates (Supplementary Fig. 1).

**Figure 5:**
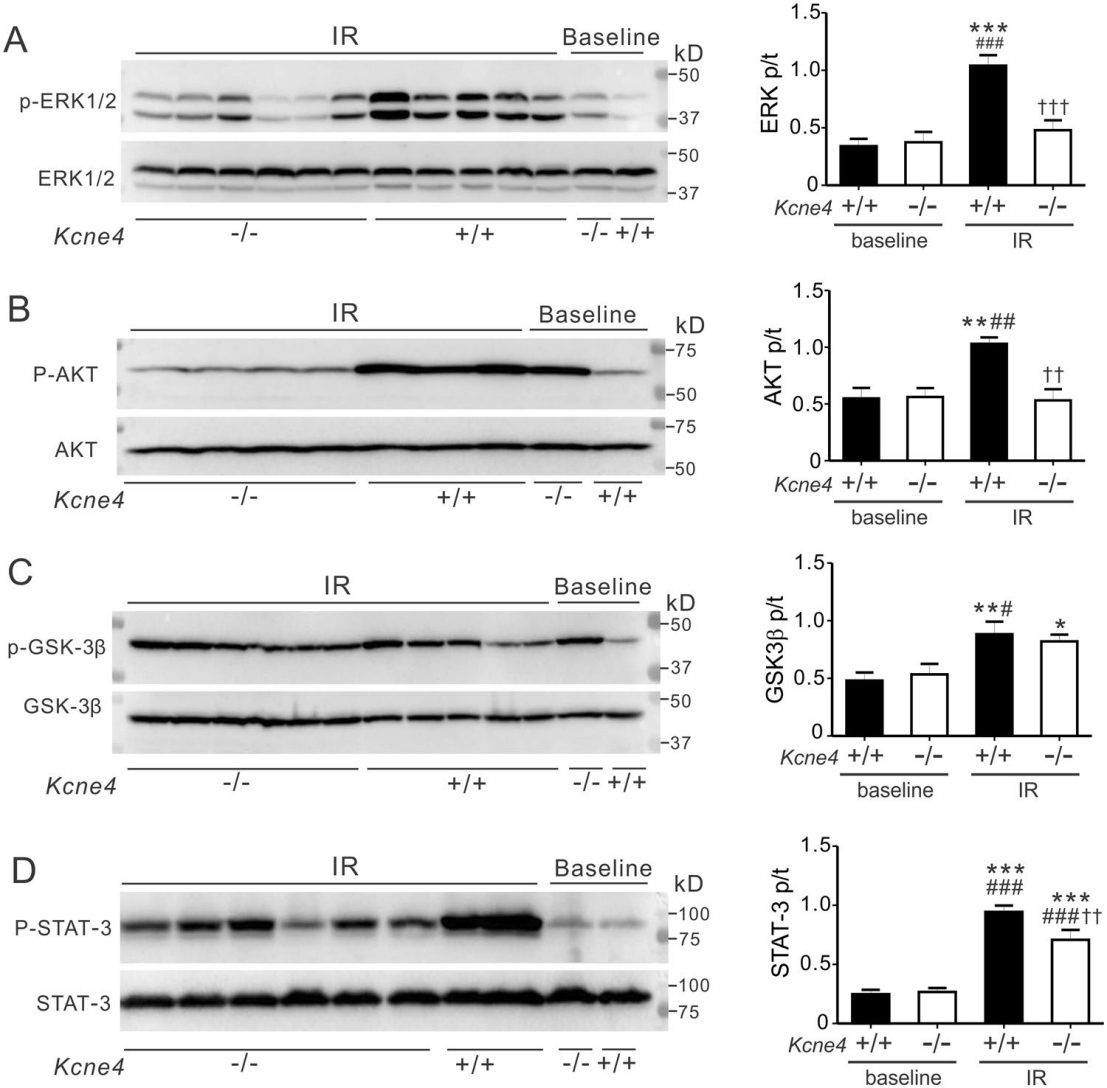
Kcne4 deletion impairs post-IR ventricular RISK/SAFE pathway induction in male mice. (**A**) *Left*, representative western blots of phosphorylated (p) ERK1/2 and total (t) ERK1/2 isolated from baseline and post-IRI male *Kcne4*^+/+^ and *Kcne4*^−/−^ ventricles. IR: hearts subjected to 10 min left coronary ligation followed by 20 min of reperfusion; one mouse per lane. *Right*, mean ratio of pERK/tERK band density from blots as in left; *n* = 8–12. ***P < *0.001*, compared with baseline male *Kcne4*^+/+^ mice; ^*###*^*P* < *0.001*, compared with baseline male *Kcne4*^−/−^ mice; ^†††^*P* < *0.001*, compared with male *Kcne4*^+/+^ mice post-IR (by One-way ANOVA). (**B**) *Left*, representative western blots of phospho- (p) AKT and total (t) AKT isolated from baseline and post-IR male *Kcne4*^+/+^ and *Kcne4*^−/−^ ventricles; one mouse per lane. *Right*, mean ratio of pAKT/tAKT band density from blots as in left. *n* = 8–10; **P < *0.01*, compared with baseline male *Kcne4*^+/+^ mice; ^*##*^*P* < *0.01*, compared with baseline male *Kcne4*^−/−^ mice; ^††^*P* < *0.01*, compared with male *Kcne4*^+/+^ mice post-IR (by One-way ANOVA). (**C**) *Left*, representative western blots of phospho- (p) GSK3β and total (t) GSK3β isolated from baseline and post-IR male *Kcne4*^+/+^ and *Kcne4*^−/−^ ventricles; one mouse per lane. *Right*, mean ratio of pGSK3β/tGSK3β band density from blots as in left; *n* = 7–9. ^*^P < 0.05, **P < *0.01*, compared with baseline male *Kcne4*^+/+^ mice, ^*#*^*P* < *0.05*, compared with baseline male *Kcne4*^−/−^ mice (by One-way ANOVA). (**D**) *Left*, representative western blots of phospho- (p) STAT-3 and total (t) STAT-3 isolated from baseline and post-IR male *Kcne4*^+/+^ and *Kcne4*^−/−^ ventricles; one mouse per lane. *Right*, mean ratio of pSTAT-3/tSTAT-3 band density from blots as in left; *n* = 8–9. ***P < *0.001*, compared with baseline male *Kcne4*^+/+^ mice, ^*###*^*P* < *0.001*, compared with baseline *Kcne4*^−/−^ mice, ^††^*P* < *0.01*, compared with male *Kcne4*^+/+^ mice post-IR (by One-way ANOVA).

**Figure 6:**
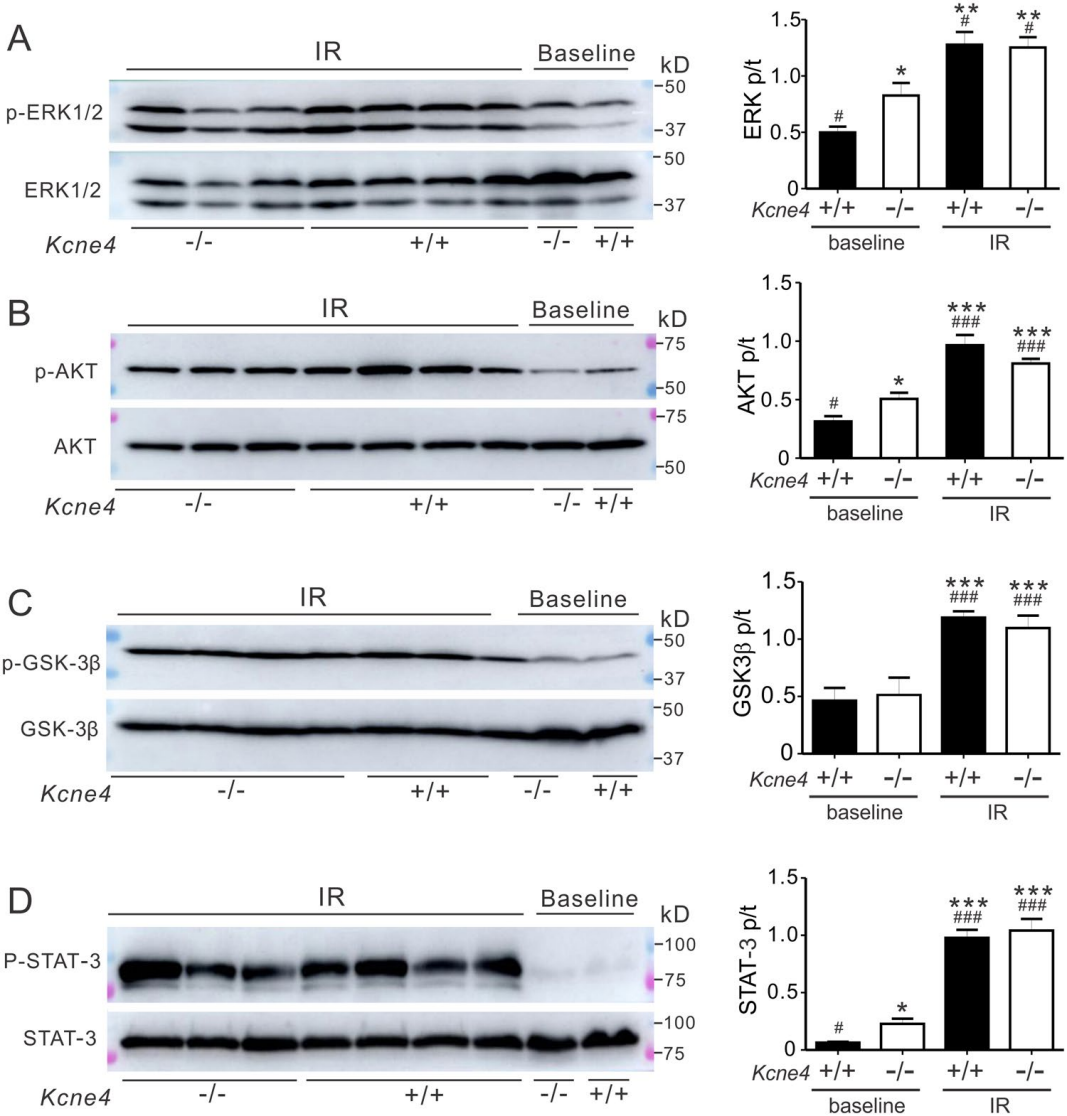
Kcne4 deletion does not impair post-IR ventricular RISK/SAFE pathway induction in female mice. (**A**) *Left*, representative western blots of phosphorylated (p) ERK1/2 and total (t) ERK1/2 isolated from baseline and post-IRI female *Kcne4*^+/+^ and *Kcne4*^−/−^ ventricles. IR: hearts subjected to 10 min left coronary ligation followed by 20 min of reperfusion; one mouse per lane. *Right*, mean ratio of pERK/tERK band density from blots as in left; *n* = 5–8. *P < 0.05, **P < *0.01*, compared with baseline female *Kcne4*^+/+^ mice; ^*#*^*P* < *0.05*, compared with baseline female *Kcne4*^−/−^ mice (by One-way ANOVA). (**B**) *Left*, representative western blots of phospho- (p) AKT and total (t) AKT isolated from baseline and post-IR female *Kcne4*^+/+^ and *Kcne4*^−/−^ ventricles; one mouse per lane. *Right*, mean ratio of pAKT/tAKT band density from blots as in left; *n* = 7–8. *P < 0.05, ***P < *0.001* compared with baseline female *Kcne4*^+/+^ mice; ^*#*^*P* < *0.05*,^*###*^*P* < *0.001* compared with baseline female *Kcne4*^−/−^ mice (by One-way ANOVA). (**C**) *Left*, representative western blots of phospho- (p) GSK3β and total (t) GSK3β isolated from baseline and post-IR female *Kcne4*^+/+^ and *Kcne4*^−/−^ ventricles; one mouse per lane. *Right*, mean ratio of pGSK3β/tGSK3β band density from blots as in left; *n* = 6–9. ***P < *0.001* compared with baseline female *Kcne4*^+/+^ mice; ^*###*^*P* < *0.001* compared with baseline female *Kcne4*^−/−^ mice (by One-way ANOVA). (**D**) *Left*, representative western blots of phospho- (p) STAT-3 and total (t) STAT-3 isolated from baseline and post-IR female *Kcne4*^+/+^ and *Kcne4*^−/−^ ventricles; one mouse per lane. *Right*, mean ratio of pSTAT-3/tSTAT-3 band density from blots as in left; *n* = 6–8. *P < 0.05, ***P < *0.001* compared with baseline female *Kcne4*^+/+^ mice; ^*#*^*P* < *0.05*, ^*###*^*P* < *0.001* compared with baseline female *Kcne4*^−/−^ mice (by One-way ANOVA).

We also tested whether post-ischemic induction of the RISK and SAFE pathways was sex-dependently affected by *Kcne4* deletion, and found striking differences. IR increased phosphorylation of ventricular ERK1/2 (P < 0.001, Fig. 5A), AKT (P < 0.05, Fig. 5B), GSK-3β (Ser9) (P < 0.05, Fig. 5C) and STAT-3 (P < 0.001, Fig. 5D) in male *Kcne4*^+/+^ ventricles compared to male *Kcne4*^+/+^ mice at baseline, but failed to elevate the ratio of pERK1/2 to tERK1/2, and pAkt/tAkt in male *Kcne4*^−/−^ mice (P > 0.05 vs. *Kcne4*^−/−^ mice at baseline). pGSK-3β/tGSK-3β visibly increased in male *Kcne4*^−/−^ mice after IR but this did not did not reach statistical significance (P > 0.05 vs. *Kcne4*^−/−^ mice at baseline) (Fig. 5A–C). The ratio of phosphorylated to total STAT-3 in the RISK pathway doubled in male *Kcne4*^−/−^ mice post IR when compared to male *Kcne4*^−/−^ mice at baseline (P < 0.01, Fig. 5D), but this did not achieve the phosphorylation level of male *Kcne4*^+/+^ mice post-IR (P < 0.05 between genotypes after IR). In marked contrast, ERK1/2 (Fig. 6A), AKT (Fig. 6B), GSK-3β (Fig. 6C) and STAT-3 (Fig. 6D) phosphorylation was significantly increased in female mice post-IR, independent of genotype (all P > 0.05), compared to their wildtype littermates at baseline (P < 0.05, P < 0.01 or P < 0.001). Thus, male, but not female, *Kcne4*^−/−^ mice exhibit defective RISK/SAFE pathway induction in response to IR injury.

### RISK/SAFE pathway induction underlies the lower incidence of IR-induced ventricular tachyarrhythmias in female versus male Kcne4^−/−^ mice

**Figure 7:**
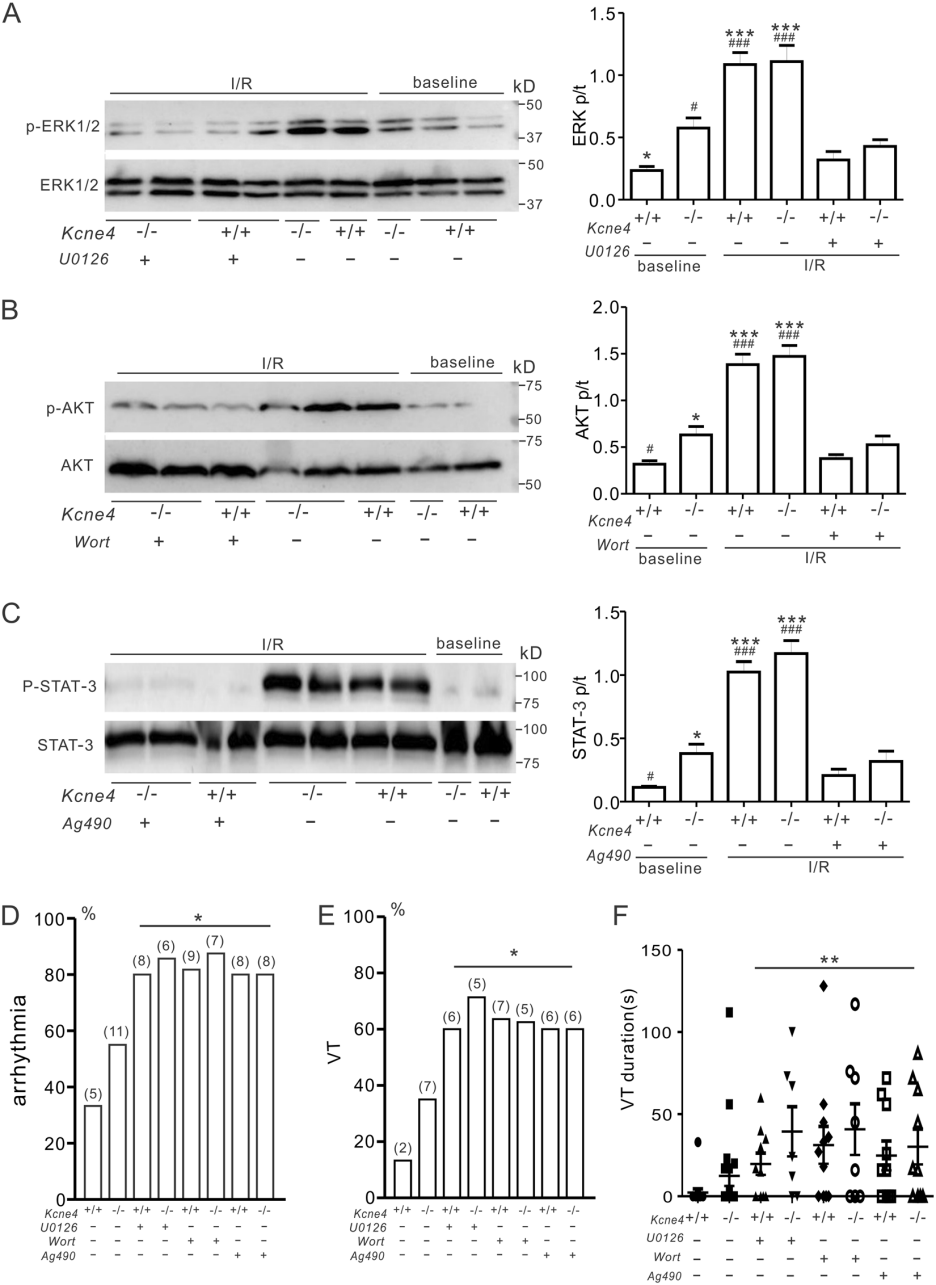
Inhibition of ERK1/2, AKT, or STAT-3 phosphorylation increases arrhythmia predisposition in female mice post-IR. (**A**) *Left*, representative western blots of phosphorylated (p) ERK1/2 and total (t) ERK1/2 isolated from baseline and post-IRI female *Kcne4*^+/+^ and *Kcne4*^−/−^ ventricles in the absence or presence of U0126. IR: hearts subjected to 10 min left coronary ligation followed by 20 min of reperfusion; one mouse per lane. *Right*, mean ratio of pERK/tERK band density from blots as in left; *n* = 5–6. *P < 0.05, ***P < *0.001*, compared with baseline female *Kcne4*^+/+^ mice; ^*#*^*P* < *0.05*, ^*###*^*P* < *0.001*, compared with baseline female *Kcne4*^−/−^ mice (by One-way ANOVA). (**B**) *Left*, representative western blots of phosphorylated (p) AKT and total (t) AKT isolated from baseline and post-IRI female *Kcne4*^+/+^ and *Kcne4*^−/−^ ventricles in the absence or presence of wortmannin (wort). IR: hearts subjected to 10 min left coronary ligation followed by 20 min of reperfusion; one mouse per lane. *Right*, mean ratio of pAKT/tAKT band density from blots as in left; *n* = 5–6. *P < 0.05, ***P < *0.001*, compared with baseline female *Kcne4*^+/+^ mice; ^*#*^*P* < *0.05*, ^*###*^*P* < *0.001*, compared with baseline female *Kcne4*^−/−^ mice (by One-way ANOVA). (**C**) *Left*, representative western blots of phosphorylated (p) STAT-3 and total (t) STAT-3 isolated from baseline and post-IRI female *Kcne4*^+/+^ and *Kcne4*^−/−^ ventricles in the absence or presence of Ag490. IR: hearts subjected to 10 min left coronary ligation followed by 20 min of reperfusion; one mouse per lane. *Right*, mean ratio of pSTAT-3/tSTAT-3 band density from blots as in left; *n* = 5–6. *P < 0.05, ***P < *0.001*, compared with baseline female *Kcne4*^+/+^ mice; ^*#*^*P* < *0.05*, ^*###*^*P* < *0.001*, compared with baseline female *Kcne4*^−/−^ mice (by One-way ANOVA). (**D**) Quantification of cardiac arrhythmia incidence during post-ischemia reperfusion in female *Kcne4*^+/+^ and *Kcne4*^−/−^ mice (*n* = 7–20). Numbers of mice exhibiting each arrhythmia category are indicated in parentheses. Values for female *Kcne4*^+/+^ and *Kcne4*^−/−^ hearts before inhibitor administration are repeated from Fig. 3A for comparison. Wort, wortmannin. *P < *0.05* compared with baseline female *Kcne4*^+/+^ mice. (**E**) Quantification of ventricular tachycardia (VT) incidence during post-ischemia reperfusion in female *Kcne4*^+/+^ and *Kcne4*^−/−^ mice (*n* = 7–20). Numbers of mice exhibiting each arrhythmia category are indicated in parentheses. Values for female *Kcne4*^+/+^ and *Kcne4*^−/−^ hearts before inhibitor administration are repeated from Fig. 3A for comparison. Wort, wortmannin. *P < *0.05* compared with baseline female *Kcne4*^+/+^ mice. (**F**) Mean VT durations for female *Kcne4*^+/+^ and *Kcne4*^−/−^ mice (*n* = 7–20). Mice without VT were indicated as 0 s duration. Values for female *Kcne4*^+/+^ and *Kcne4*^−/−^ hearts before inhibitor administration are repeated from Fig. 3A for comparison. Wort, wortmannin. **P < *0.01*, vs. baseline female *Kcne4*^+/+^ mice.

We next tested whether enhanced RISK/SAFE pathway induction was causally linked to the reduced IR susceptibility in female versus male *Kcne4*^−/−^ mice, by quantifying the effects on post-IR arrhythmia incidence of pharmacological inhibitors of ERK1/2 (U0126), AKT (wortmannin) and STAT3 (Ag490). As expected, pretreatment with each of the inhibitors prevented activation of their respective targets post-IR injury, verifying activity of the inhibitors (Fig. 7A–C). Strikingly, each of the inhibitors also increased post-IR arrhythmia incidence and severity in female mice (regardless of genotype), to levels similar to those of male *Kcne4*^−/−^ mice (Fig. 7D–F; compare with Fig. 3). Thus, the lower IR-induced susceptibility of female versus male *Kcne4*^−/−^ mice was dependent on RISK/SAFE pathway induction in females.

### Impaired RISK/SAFE pathway induction and predisposition to IR-induced ventricular tachyarrhythmias in male Kcne4^−/−^ mice is testosterone-dependent

**Figure 8:**
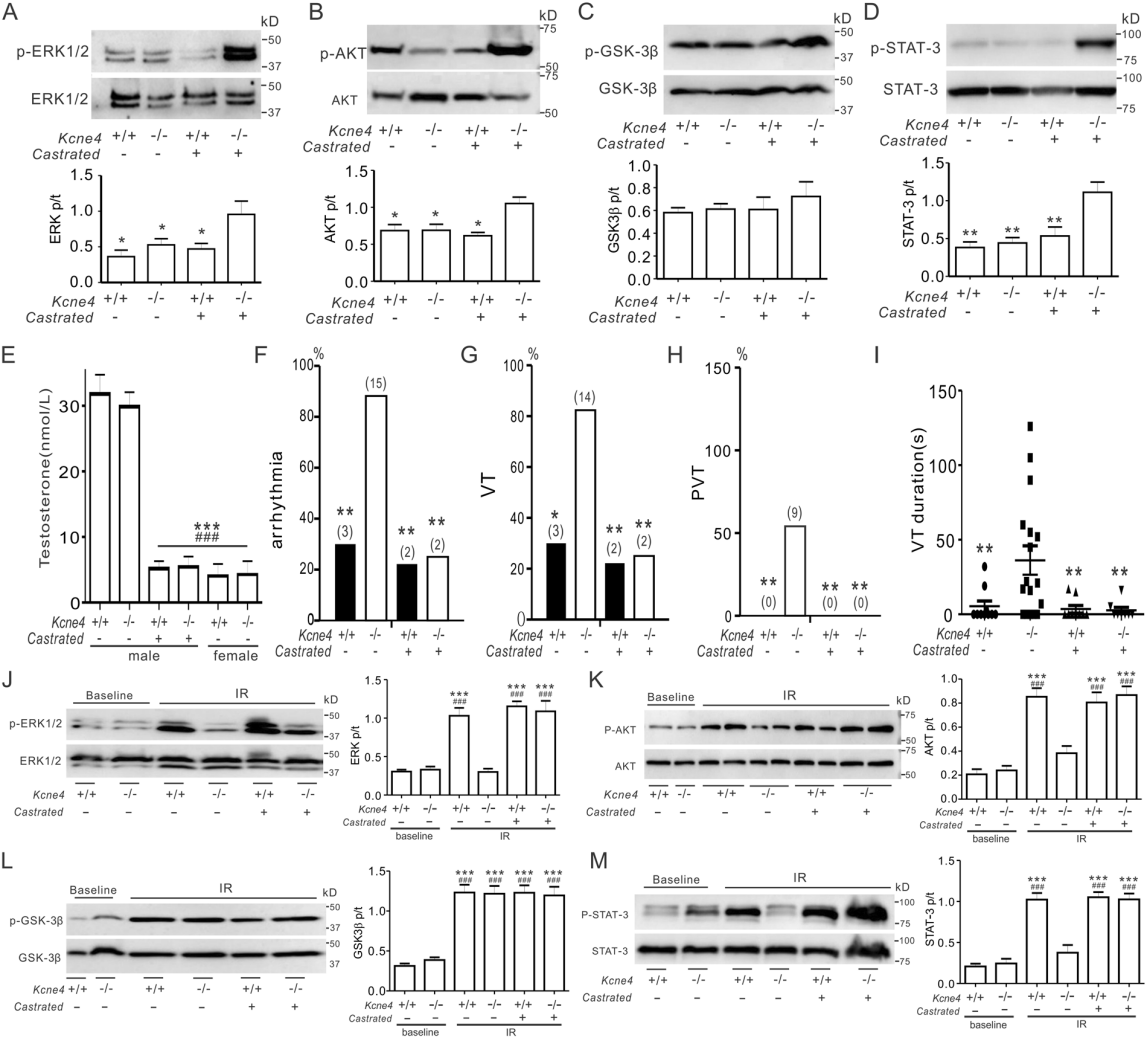
Castration decreases predisposition to severe IR ventricular arrhythmias in male Kcne4^−/−^ mice. (**A–D**) ***Upper***, representative western blots of phosphorylated (p) ERK1/2 and total (t) ERK1/2 (**A**), phospho- (p) AKT and total (t) AKT (**B**), phospho- (p) GSK3β and total (t) GSK3β (**C**), phospho- (p) STAT-3 and total (t) STAT-3 (**D**), isolated from castrated and non-castrated male *Kcne4*^+/+^ and *Kcne4*^−/−^ left ventricles. ***Lower****,* mean ratio of pERK/tERK, pAKT/AKT, pGSK3β/GSK3β, pSTAT-3/STAT-3 band densities from blots as in ***upper***; *n* = 5. *P < 0.05, **P < *0.01*, compared with castrated male *Kcne4*^−/−^ mice (by one-way ANOVA). (**E**) Bar graph showing serum concentrations of total testosterone in male (castrated and non-castrated) and female *Kcne4*^+/+^ and *Kcne4*^−/−^ mice at baseline; *n* = 6–7 each group. ***P < *0.01*, compared with non-castrated male *Kcne4*^+/+^ mice, ^*###*^*P* < *0.001*, compared with non-castrated male *Kcne4*^−/−^ mice (by One-way ANOVA). (**F**–**H**) Quantification of incidence of cardiac arrhythmia (**F**), ventricular tachycardia (VT) (**G**) and polymorphic VT (PVT) **(H)** during post-ischemia reperfusion in castrated and non-castrated male *Kcne4*^+/+^ and *Kcne4*^−/−^ mice (*n* = 8–17). Numbers of mice per category are indicated in parentheses. Values for male *Kcne4*^+/+^ and *Kcne4*^−/−^ hearts before castration are repeated from Fig. 3A for comparison. *P < 0.05, **P < *0.01* compared with non-castrated male *Kcne4*^−/−^ mice. (**I**) Mean VT durations for castrated and non-castrated male *Kcne4*^+/+^ and *Kcne4*^−/−^ mice post IR (*n* = 8–17). Mice without VT were indicated as 0 s duration. Values for male *Kcne4*^+/+^ and *Kcne4*^−/−^ hearts before castration are repeated from Fig. 3A for comparison. **P < *0.01*, vs. non-castrated male *Kcne4*^−/−^ mice (by one-way ANOVA). (**J**–**M**) ***Left***, representative western blots of phosphorylated ERK1/2 (p) and ERK1/2 (t) **(J)**, phospho- (p) AKT and total (t) AKT**(K)**, phospho- (p) GSK3β and total (t) GSK3β **(L)**, phospho- (p) STAT-3 and total (t) STAT-3 **(M)**, isolated from baseline and post-IRI castrated and non-castrated male *Kcne4*^+/+^ and *Kcne4*^−/−^ left ventricles. ***Right****,* mean ratio of pERK/tERK, pAKT/AKT, pGSK3β/GSK3β, pSTAT-3/STAT-3 band densities from blots as in ***Left***; *n* = 5. ***P < *0.001*, compared with non-castrated male *Kcne4*^+/+^ mice, ^*###*^*P* < *0.001*, compared with non-castrated male *Kcne4*^−/−^ mice (by One-way ANOVA).

Finally, we investigated the mechanism underlying the sex-dependence of RISK/SAFE pathway induction in *Kcne4*^−/−^ mice, hypothesizing that it was DHT-dependent. Castration of male mice (which reduced serum DHT levels 6-fold regardless of genotype; P < 0.001; Fig. 8E) permitted the baseline activation of ERK1/2, AKT and STAT3 in response to *Kcne4* deletion (Fig. 8A–D), which was absent in non-castrated male mice but present in females (Fig. 4). Strikingly, castration also completely ablated *Kcne4* deletion-dependent susceptibility of male mice to IR-induced ventricular arrhythmias (Fig. 8E–I). Finally, castration also eliminated the sex-dependent impairment of post-IR RISK/SAFE pathway induction (Fig. 8J–M) that we had observed in non-castrated male mice (Fig. 5).

## Discussion

Human, cardiac-expressed *KCNE* genes are well known for their association with cardiac arrhythmias including long QT syndrome^31–41^, but the ubiquitous and dynamic expression of KCNE proteins may ensure that pathologies associated with their dysfunction are multifactorial^5^. In various studies of *Kcne* gene-deleted mice, we and others have shown that due to their often ubiquitous expression outside the heart, *Kcne* gene disruption can lead to systemic as well as cardiac-specific defects, with complex interactions between the two^19,20,22,42^. Some of this complexity is now beginning to be recognized in human syndromes associated with *KCNE* gene variants^43–46^. The multifactorial disease states associated with *Kcne* disruption are particularly significant when one considers that SCD is thought to require an electrical substrate, an ischemic substrate, and a trigger. Deletion of the *Kcne2* gene in mice can both provide the electric substrate for arrhythmogenesis by directly disrupting ventricular myocyte repolarization, and can also provide an ischemic substrate by causing atherosclerosis, diabetes, fatty liver and structural heart disease. However, paradoxically, *Kcne2* deletion also leads to baseline induction of cardioprotective pathways, which can limit damage when the mice are challenged with an ischemic insult^22^.

We previously found that KCNE4 is a DHT-regulated determinant of cardiac excitability in mice, and that *Kcne4* deletion impairs ventricular repolarization in an age and sex-dependent manner, with QTc prolongation only occurring similarly in both males and females after female menopause. The QTc prolongation occurs because the normal role of *Kcne4* in mouse heart is to augment the *I*_to_ and *I*_K,slow_ currents by upregulating Kv4.3 and Kv1.5 activity^11^. Here, we again found similar QTc prolongation in aging male and female *Kcne4*^−/−^ mice, but unexpectedly discovered that despite this, aging males were still more predisposed than aging females to *Kcne4*-linked arrhythmias, when challenged with an imposed IR injury. Using pharmacological RISK/SAFE component antagonists and castration, we discovered that the greater predisposition to IR-induced arrhythmias in male mice arose from testosterone-dependent impairment of RISK/SAFE cardioprotective pathway induction.

In addition to IR-induced RISK/SAFE pathway induction, the increased baseline phosphorylation of proteins in the RISK pathway may produce a ‘cardiac preconditioning’-like phenomenon in *Kcne4*^−/−^ female mice, remodeling the heart in a manner that better prepares it for an acute ischemic event in the form of reperfusion-induced arrhythmia. We observed a similar phenomenon in *Kcne2*^−/−^ mice^22^, although in that study we did not compare sexes. Our new data support a novel arrhythmogenic mechanism in which loss of a DHT-regulated ion channel subunit sex-specifically affects cardioprotective pathway induction. Both RISK and SAFE pathways were activated at baseline in female and castrated male *Kcne4*^−/−^ mice, but, interestingly, the canonical GSK-3β phosphorylation was not, suggesting cross-talk between the two pathways should be a topic of future study. Similarly, the novel idea that disruption of a potassium channel β subunit can sex-specifically induce versus impair crucial cardioprotective signaling cascades is worth further exploration because of its implications for sex differences in predisposition to myocardial infarction, and SCD.

### Limitations and Conclusions

In summary, we have discovered that *Kcne4* deletion constitutively activates cardioprotective pathways in female mice such that they are protected from the arrhythmogenic effects of a brief but stringent IR injury in the postmenopausal period. In contrast, *Kcne4* deletion in age-matched males does not precondition; it impairs post-ischemic RISK/SAFE pathway induction; and it predisposes to IR injury-induced ventricular arrhythmias. This sex difference is prevented by castration. To our knowledge, this is the first reported example of a monogenic basis for sex-specific differences in RISK/SAFE pathway induction, and the results may have implications for therapeutic approaches involving these pathways.

One limitation of our study is that, perhaps surprisingly, human *KCNE4* gene variants have not yet been linked to ventricular arrhythmias, although a *KCNE4* polymorphism (E145D, short isoform numbering) is associated with increased risk of AF^47^. Thus, we do not yet know the specific role of KCNE4 in human ventricles, despite it reportedly being the highest expressed ventricular KCNE subunit^7,8^. Another limitation is that we have not discovered the specific molecular basis for *Kcne4* deletion-induced baseline RISK/SAFE pathway activation in the female and castrated male mice. We eliminated several potential mechanisms, including electrical substrate (Fig. 1), structural heart disease and fibrosis (Supplementary Fig. 1). KCNE4 is expressed in the apical membrane of renal intercalated cells, where it is thought to regulate BK channels and possibly modulate urinary potassium secretion, but the sex-specificity of this is not known^14^. KCNE4 also regulates KCNQ4 in rat and mouse blood vessels, and in young adult mice *Kcne4* deletion is differentially manifested, with males but not females exhibiting increased contractility in response to the α-adrenoceptor agonist methoxamine, but decreased responses to the Kv7.2–7.5 channel activator ML213. In contrast, *Kcne4* deletion strongly decreased isoprenaline-induced vasorelaxation in both male and female mice (22). One possibility is that mismatches in vascular reactivity lead to sex-specific induction of cardiac RISK/SAFE pathways, in females but not non-castrated males. To further delineate the molecular mechanisms underlying our findings, a future approach could involve creating tissue-specific *Kcne4*^−/−^ mice to identify whether *Kcne4* deletion from the heart, vasculature, kidneys or other tissues testosterone-dependently predisposes to ventricular tachyarrhythmias in the context of IR injury.

## Electronic supplementary material

Supplementary Information
