# Supplementary material for: Kcne4 deletion sex-specifically predisposes to cardiac arrhythmia via testosterone-dependent impairment of RISK/SAFE pathway induction in aged mice
Source: Sci Rep. 2018 May 29;8:8258. doi: 10.1038/s41598-018-26599-8 (PMC5974354; doi:10.1038/s41598-018-26599-8)

Kcne4 deletion sex-specifically predisposes to cardiac arrhythmia via testosterone-dependent impairment of RISK/SAFE pathway induction in aged mice

Zhaoyang Hu[1]*, Wei Wei[2], Leng Zhou[2], Mou Chen[1], Geoffrey W. Abbott[3]*

[1] Laboratory of Anesthesiology & Critical Care Medicine, Translational Neuroscience Center, West China Hospital, Sichuan University, Chengdu, Sichuan, China

[2] Department of Anesthesiology, West China Hospital, Sichuan University, Chengdu, Sichuan, China

[3] Bioelectricity Laboratory, Dept. of Physiology and Biophysics, School of Medicine, University of California, Irvine, CA, USA

* To whom correspondence should be addressed: Dr. Geoffrey W. Abbott, abbottg@uci.edu; or Dr. Zhaoyang Hu, zyhu@hotmail.com

Keywords: cardiac arrhythmia; ischemia/reperfusion injury; potassium channel; Long QT syndrome; sudden cardiac death

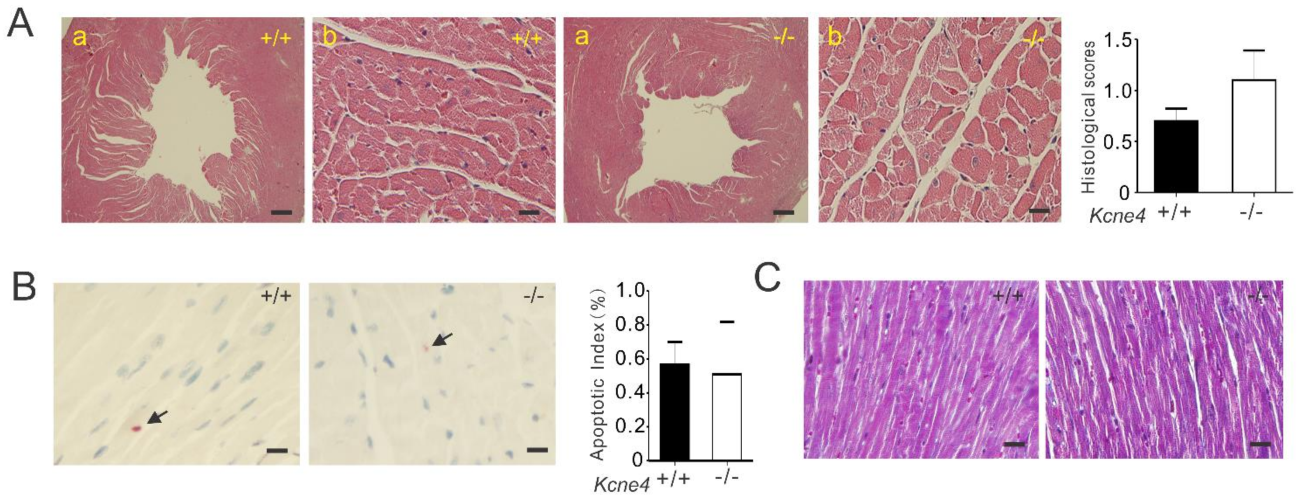

Supplementary Figure 1. *Kcne4* deletion does not cause structural heart disease in male mice.

A. Left: representative histological H&E-stained micrographs of cardiac section from male $Kcne4^{+/+}$ and $Kcne4^{-/-}$ mice as indicated, representative of n = 5 mice per genotype. Panel (a) scale bars: 100 mm; panel (b) scale bars, 10 mm. Right: morphological evaluation of both genotypes (n = 5 mice per genotype).

B. ***Left***: representative TUNEL-stained heart sections of male mice of both genotypes at baseline.
Arrows indicate TUNEL-positive nuclei(red). Scale bars, 10 mm. Right: graph showing the averaged percentage of TUNEL-positive cells (n=5, each genotype). *NS,* Non significant difference between genotype.

C. Masson trichrome–stained sections from male mice of both genotypes at baseline. Scale bars, 10 mm.

Raw western blots

marker information:

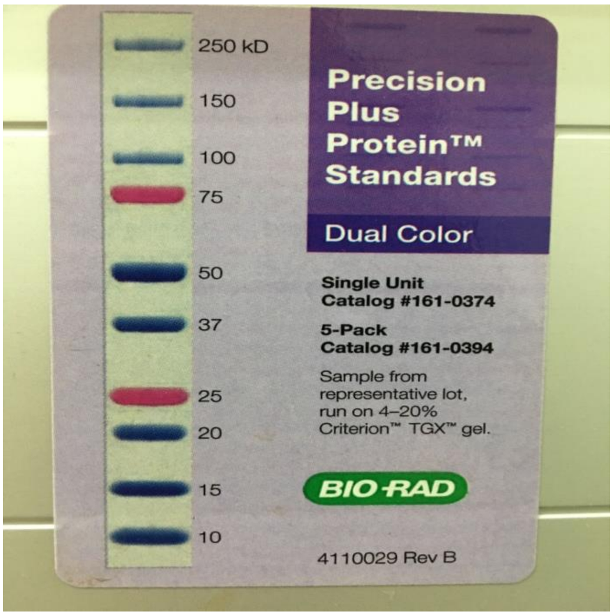

Figure 4B

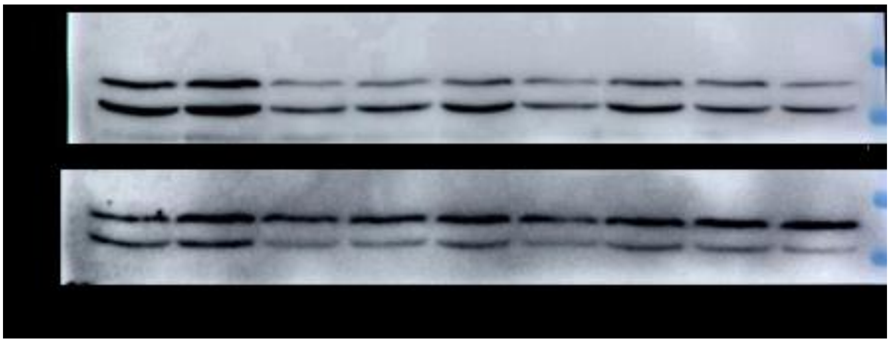

Figure 4C

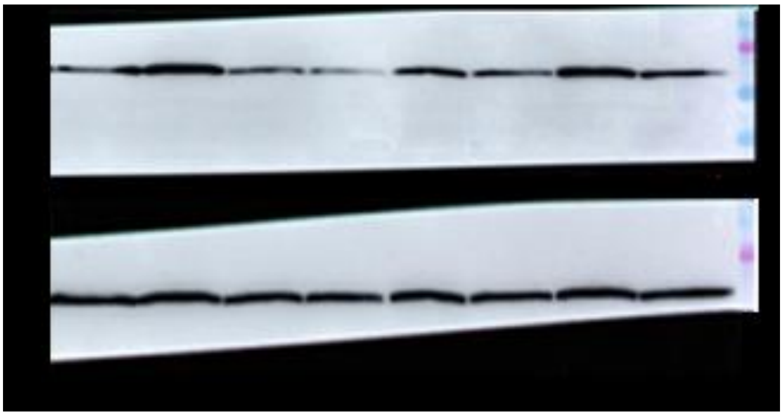

Figure 4D

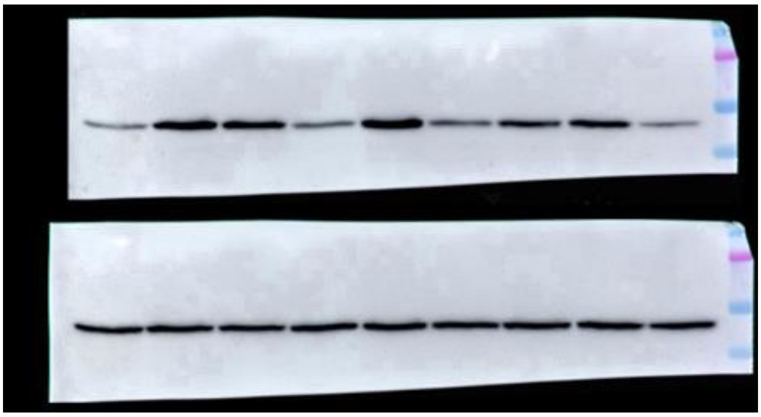

Figure 4E

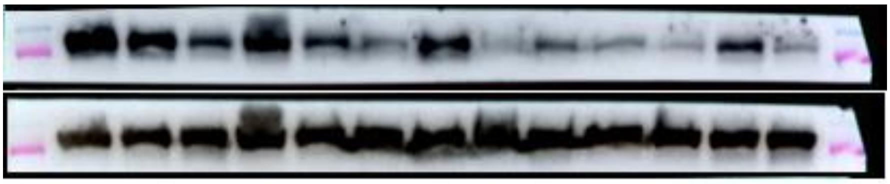

Figure5A

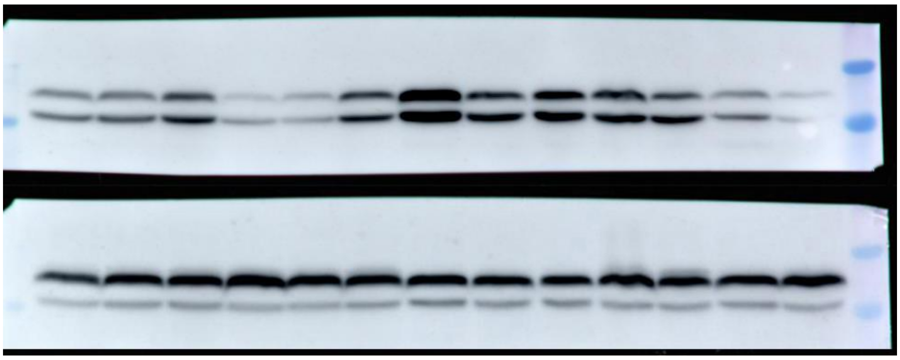

Figure5B

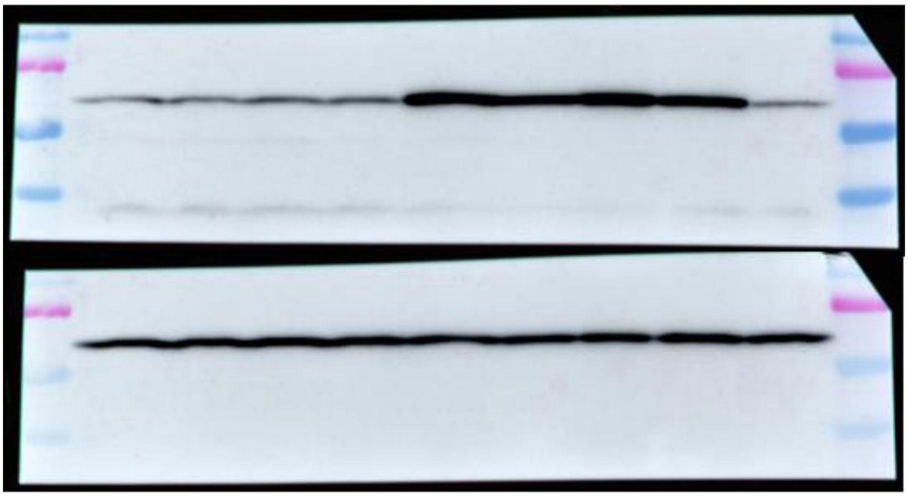

Figure5C

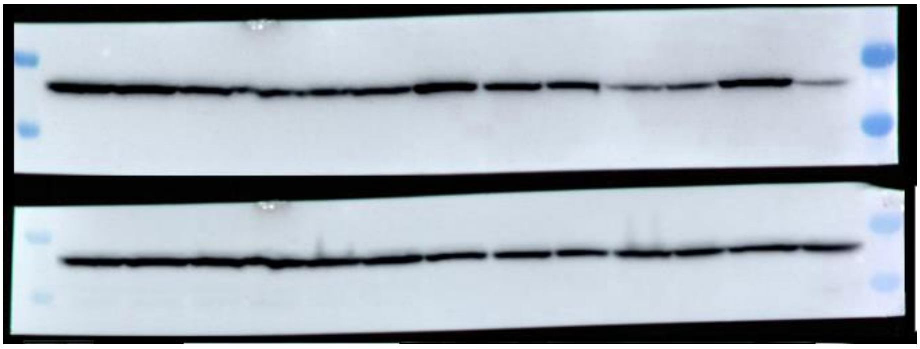

Figure5D

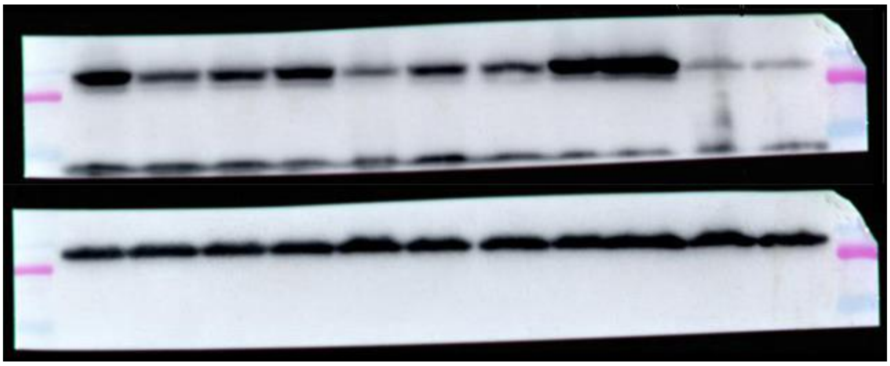

Figure 6A

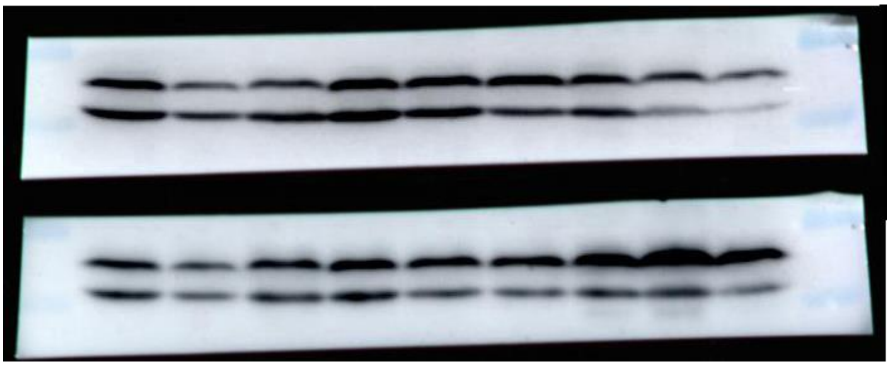

Figure 6B

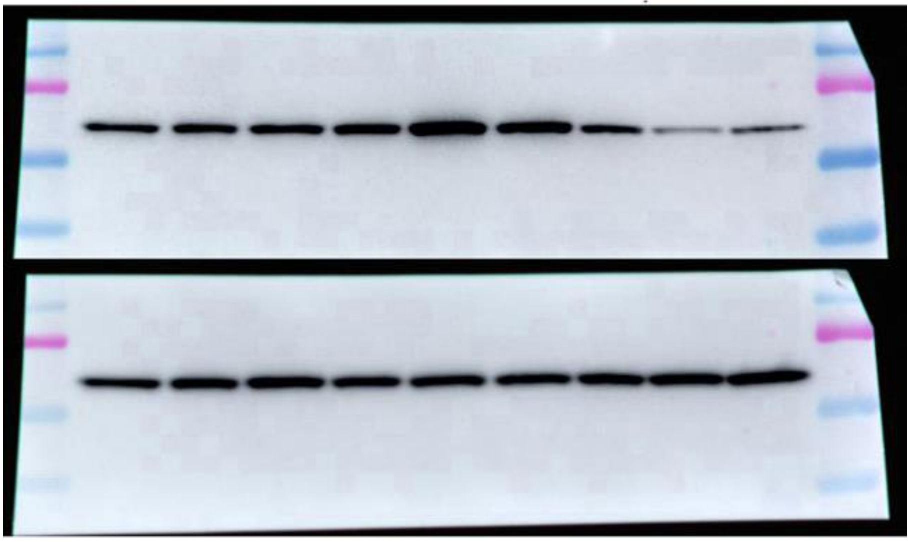

Figure 6C

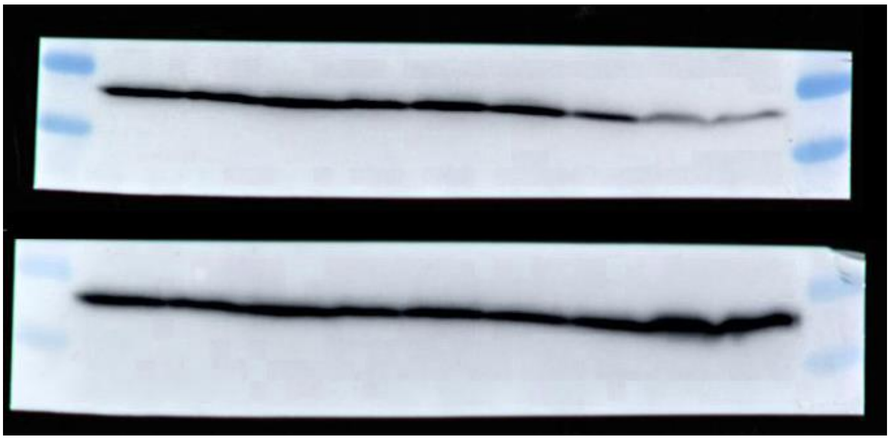

Figure 6D

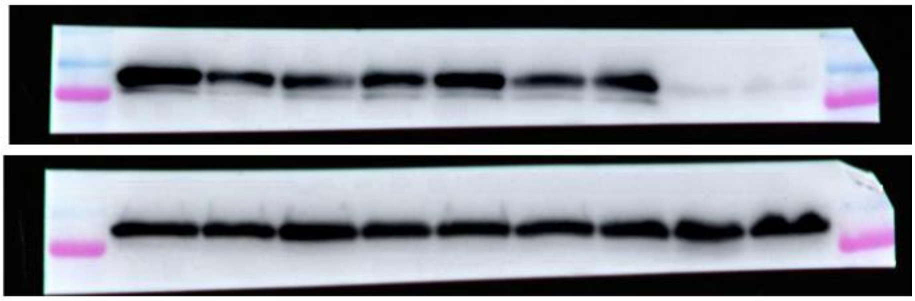

Figure 7A

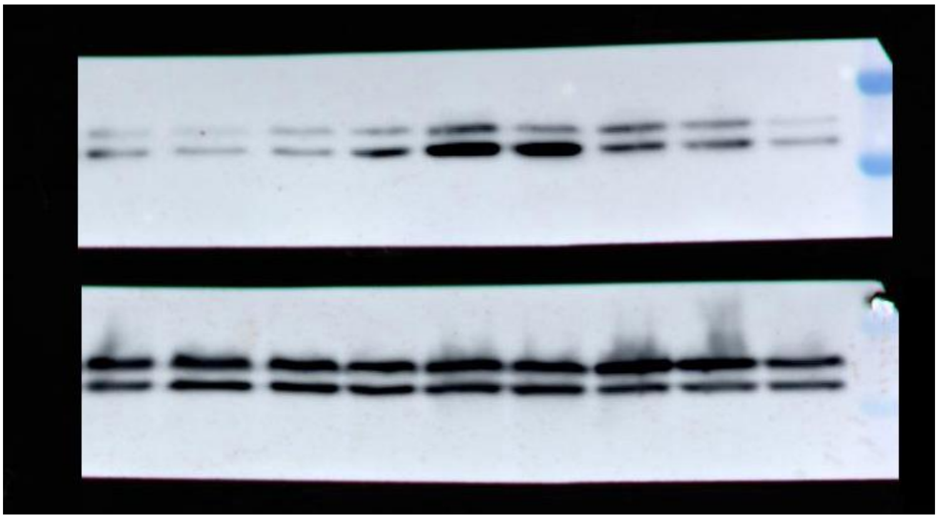

Figure 7B

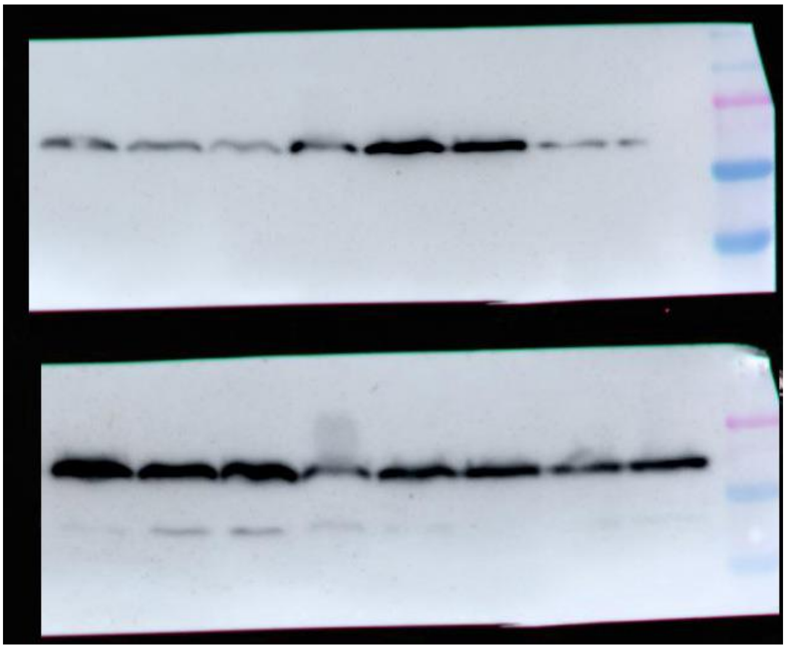

Figure 7C

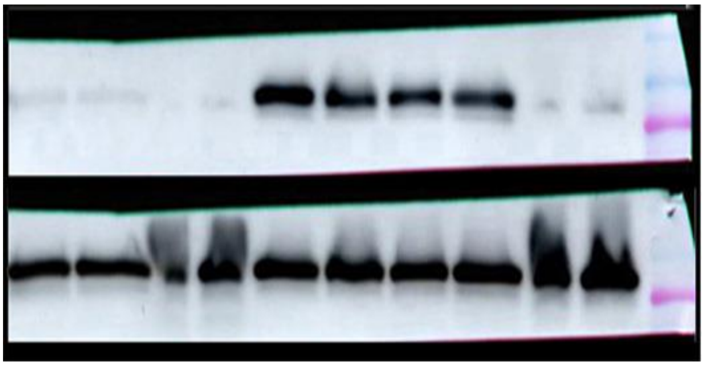

Figure8A

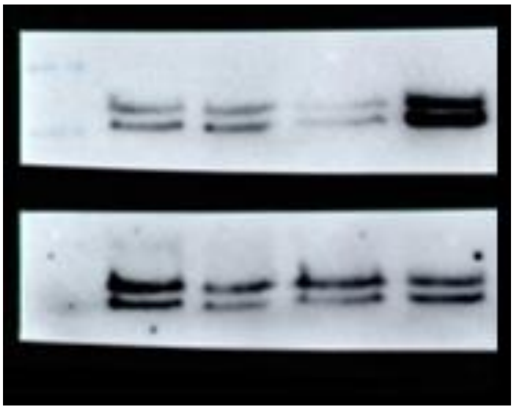

Figure 8B

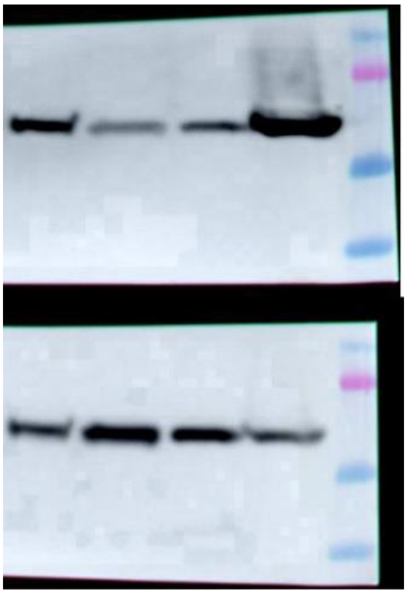

Figure 8C

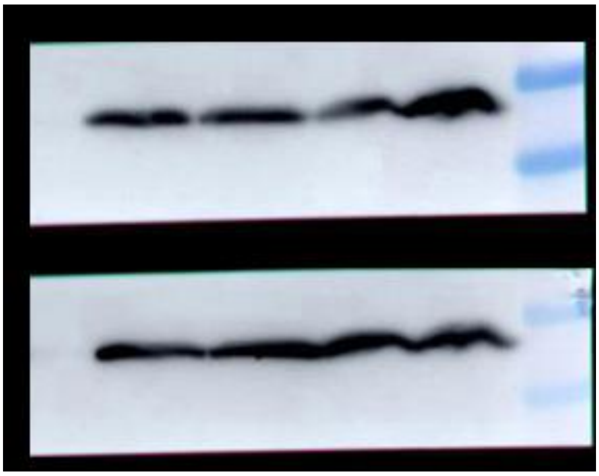

Figure 8D

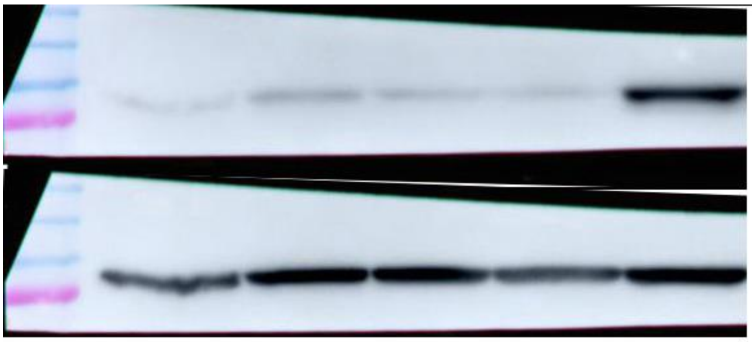

Figure 8I

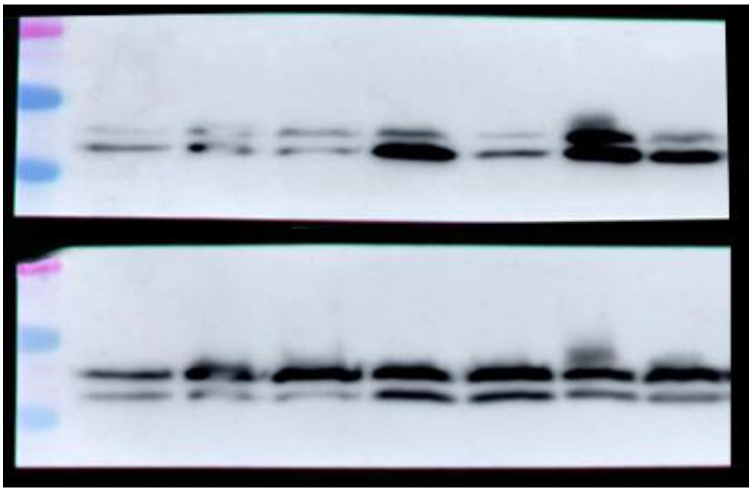

Figure 8J

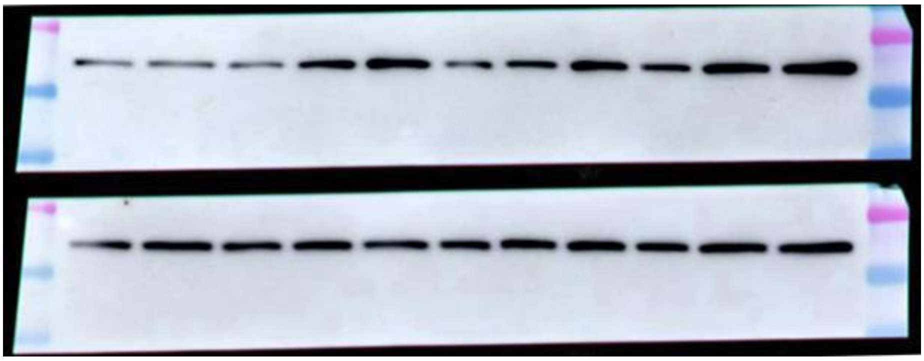

Figure 8K

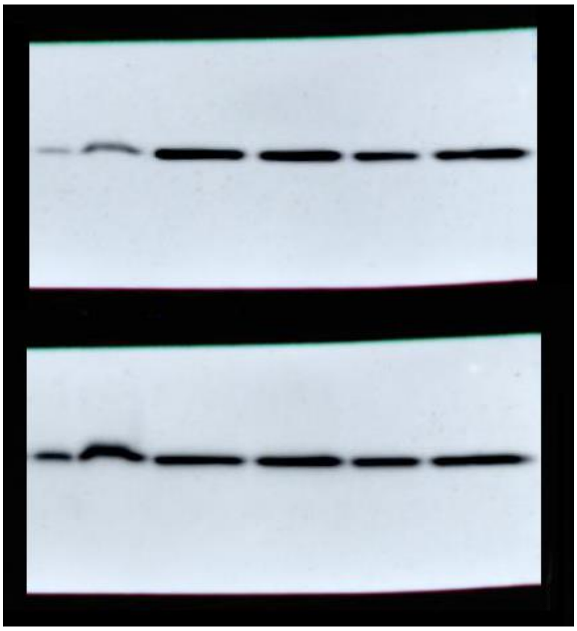

Figure 8L

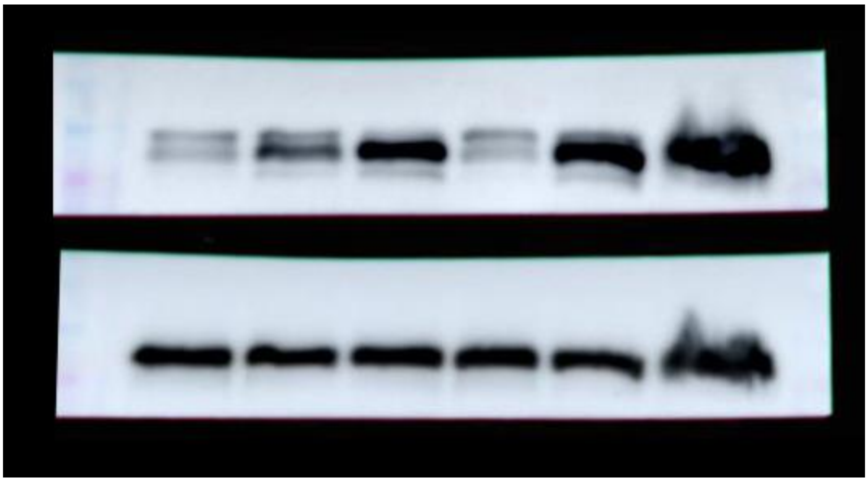

Supplement: Supplementary file 1 — Supplementary Information [file 41598_2018_26599_MOESM1_ESM.pdf]
